# Investigating Attraction and Retention of Staff Within Public Mental Health Services in Victoria, Australia: Protocol for a Mixed Methods Study

**DOI:** 10.2196/48855

**Published:** 2023-10-31

**Authors:** Kaitlyn Crocker, Inge Gnatt, Darren Haywood, Ravi Bhat, Ingrid Butterfield, Anoop Raveendran Nair Lalitha, Ruby Bishop, David J Castle, Zoe M Jenkins

**Affiliations:** 1 Department of Mental Health St Vincent's Hospital Melbourne Fitzroy Australia; 2 Melbourne Neuropsychiatry Centre The University of Melbourne Carlton Australia; 3 Centre for Mental Health and Brain Sciences Swinburne University of Technology Melbourne Australia; 4 Turner Institute for Brain and Mental Health School of Psychological Science Monash University Clayton Australia; 5 Human Performance Research Centre, INSIGHT Research Institute, School of Sport, Exercise and Rehabilitation University of Technology Sydney NSW Australia; 6 Department of Psychiatry The University of Melbourne Melbourne Australia; 7 Department of Rural Health The University of Melbourne Shepparton Australia; 8 Goulburn Valley Area Mental Health Service Goulburn Valley Health Shepparton Australia; 9 HER Centre Australia Monash University Clayton Australia; 10 Department of Psychiatry Cabrini Health Malvern Australia; 11 Grampians Mental Health & Wellbeing Services Grampians Health Ballarat Australia; 12 Mercy Mental Health & Wellbeing Services Mercy Health Melbourne Australia; 13 School of Psychological Sciences University of Tasmania Tasmania Australia; 14 Centre for Mental Health Services Innovation Statewide Mental Health Services Tasmania Australia

**Keywords:** mental health personnel, career choice, recruitment, retention, turnover intention, public mental health, mental health, workforce, challenges, attraction, retention of staff, staff retention, human resources, human resourcing, HR, hire, hiring, new hire, onboarding, orientation, workforce

## Abstract

**Background:**

A large proportion of Australians are affected by mental illness each year, and treatment gaps are well known. To meet current and future demands and enable access to treatment that is safe, effective, and acceptable, a robust and sustainable mental health workforce is required. Factors reported to attract people to work within the mental health sector include aspiring to help others, having an interest in mental health and human behavior, the desire to make a difference and do something worthwhile, personal lived experience, recognition, and value of discipline-specific roles. However, despite the various reasons people enter the public mental health workforce, recruitment and retention continue to be ongoing challenges. To date, there has been limited investigation into understanding which factors are most relevant to the current Victorian workforce. Furthermore, a comparison to health care workers outside of mental health is also needed to better understand the specific needs of staff within the mental health sector.

**Objective:**

This study aims to explore factors related to attraction, recruitment, and retention of the public mental health workforce in Victoria, Australia.

**Methods:**

The study is a multisite, mixed methods cross-sectional study to be conducted at 4 public hospital services within Victoria, Australia: 2 in metropolitan and 2 in regional or rural locations. Current, previous, and nonmental health workers will be asked to complete a 20-25–minute web-based survey, which is developed based on previous research and offered participation in an optional 30-60–minute semistructured interview to examine personal experiences and perceptions. Both aspects of the project will examine factors related to attraction, recruitment, and retention in the public mental health workforce. Differences between groups (ie, current, past, and nonmental health workers), as well as location, discipline, and health setting will be examined. Regression analyses will be performed to determine the factors most strongly associated with retention (ie, job satisfaction) and turnover intention. Qualitative data will be transcribed verbatim and thematically analyzed to identify common themes.

**Results:**

As of May 2023, we enrolled 539 participants in the web-based survey and 27 participants in the qualitative interview.

**Conclusions:**

This project seeks to build on current knowledge from within Australia and internationally to understand role and service/system-related issues of attraction, recruitment, and retention specifically within Victoria, Australia. Seeking up-to-date information from across the health workforce may provide factors specific to mental health by illuminating any differences between mental health workers and health care workers outside of mental health. Furthermore, exploring motivators across health care disciplines and locations to enter, stay in, or leave a role in public mental health settings will provide valuable information to support how the sector plans and develops strategies that are fit for purpose.

**International Registered Report Identifier (IRRID):**

DERR1-10.2196/48855

## Introduction

### Background

One-fifth of Australians experience mental illness each year, and treatment gaps both in type and quality are well known [1,2]. Within Australia, there was a reported treatment gap of 45% for people with major depression, while the average duration of untreated psychosis was reported to be 6 months [3]. Moreover, among adolescents who perceived the need for mental health (MH) treatment, only 53% received it [4]. To enable access to treatment and care that is safe, effective, and acceptable to patients and their families, a robust and sustainable MH workforce is required. The Royal Commission into Victoria’s Mental Health System described historical underinvestment and increasing demand, alongside updated models of care driven by the promotion of well-being rather than an illness focus, resulting in an outdated and crisis-driven service that is ill-equipped to respond adequately to people living with mental illness, their families, and caregivers [5]. A critical issue is attracting and retaining staff in the MH workforce [6]. Evidence suggests that compared to other departments (eg, general medical and surgical), the MH workforce experiences higher rates of staff turnover and lower retention, although there is a paucity of exact statistics [7]. In order to understand the factors related to the recruitment and retention of MH workers, it is important to appreciate what attracts individuals to the MH workforce, what factors impact retention, and why people leave their role in public MH.

Past research has identified barriers to people choosing to enter the public MH workforce, including the stigma of working in MH, lack of opportunity for advancement, and disparity of remuneration between the public and private sector [8,9]. However, there are also several factors that attract people to work within the MH sector including aspiring to help others, having an interest in MH or behavior, the desire to make a difference and do something worthwhile, personal lived experience, recognition and value of discipline-specific roles, and being able to use skills and knowledge within their professional framework [5,10-14]. Despite the unique reasons that motivate people to work in MH, recruitment and retention of an adequate number of trained and experienced MH staff is an ongoing concern.

In Australia, as in other jurisdictions, the demand for public health services is growing, and there is a critical shortage of experienced MH professionals, particularly within the public MH sector [5]. This problem is further exacerbated in rural and remote areas, whereby there is significant maldistribution of the MH workforce [15]. During the COVID-19 pandemic, this problem became more pronounced as staff members were seconded to help with the response to COVID-19 (eg, to work in vaccination clinics), with not all returning to their previous role as vaccination clinics wound down. Compounding this issue, international border closures prevented overseas professionals from being granted entry into Australia, which has long been a pipeline for recruitment into the MH workforce.

In summary, there are significant factors that may contribute to low recruitment and retention within the public MH workforce; yet, there has been limited investigation into understanding which of these factors are most relevant in Victoria, Australia, and where differences may be in comparison to health care workers outside of MH. This lack of data and broad consultation across the workforce may result in initiatives being misplaced or failing to address problematic areas despite best efforts. Understanding the differences between MH workers and health care workers outside of MH will reveal the most pertinent factors that may be specific to MH. Furthermore, exploring what motivates people across health care disciplines to work or not work in public MH settings or decide to leave a job in public MH across both metropolitan and regional or rural workplaces can inform how the sector plans and develops strategies that are fit for purpose.

### Study Aims

This study aims to explore factors related to the attraction, recruitment, and retention of the public MH workforce in Victoria, Australia. Specifically, the study aims to identify (1) the reasons for entering the MH workforce, (2) the reasons for staying in the MH workforce, (3) the reasons for leaving the MH workforce, and (4) reasons for not entering the MH workforce.

This information will then be used to generate and provide specific strategies that will address the challenges identified in the evaluation.

## Methods

### Study Design and Setting

The study is a multisite, mixed methods cross-sectional study to be conducted at 4 public hospital sites in Victoria, Australia (2 in metropolitan and 2 in rural locations). A steering committee consisting of all investigators across the 4 sites will meet regularly to oversee all operational, procedural, and policy aspects of the project’s conduct.

All employees working within the MH department at each of the participating sites will be asked by email to complete a web-based survey. Staff from the wider hospital (ie, past MH workers or non-MH workers) at each site will also be invited to participate via a service-wide broadcast email. Past MH workers from each participating site who are no longer employed within the service will also be invited to participate by current employees forwarding information. Employees across all sites will also be invited to participate in a qualitative interview. Table 1 outlines the research framework.

**Table 1:** Research framework.

Dimensions	Attractiveness and recruitment	Retention	Method
Indicators	Reasons for joining or not entering the MH workforce	Reasons for staying in or leaving the MH workforce	N/A^a^
Phase 1	Current methods of assessment of reasons for joining or not entering the MH workforce	Current methods of assessment of reasons for remaining in or leaving the MH workforce	Preliminary interviews with managers of MH workforce
Phase 2a	Quantitative survey collection of reasons for joining or not entering the MH workforce	Quantitative survey collection of reasons for remaining in or leaving the MH workforce	Anonymous survey distribution across four sites
Phase 2b	Qualitative interview collection of reasons for joining or not entering the MH workforce	Qualitative interview collection of reasons for remaining in or leaving the MH workforce	Interviews with staff working in MH and in other departments across four sites
Phase 3	Compile a strategy for improving the attractiveness of working in public MH services	Compile a strategy for improving retention of staff working in public MH services	Propose methods to improve the attractiveness and retention of staff in MH services through data collected in Phase 2.

^a^N/A: not applicable.

### Participants

#### Inclusion Criteria

Participants who satisfy the criteria for any of the following groups will be eligible to participate in the quantitative survey and the qualitative interview or focus group: (1) staff who are currently employed within the MH department at each of the 4 study sites, (2) staff who were previously employed within the MH department at each of the 4 study sites, and (3) staff who are currently employed outside the MH department at each of the 4 study sites and have never worked within the MH department.

#### Exclusion Criteria

Other than sufficient English language ability to respond to the questionnaires and interview questions in a meaningful manner, there will be no specific exclusion criteria for this study to ensure representativeness and generalizability.

#### Sample Size Estimation and Justification

Based on the current employment records, this study aims to collect questionnaire feedback from all employees working within the MH department of each health service; however, previous such studies have shown an approximate 30% response rate, with calculations as follows: (1) metropolitan site 1 (n=135 people), (2) metropolitan site 2 (n=150 people); (3) rural site 1 (n=111 people), and (4) rural site 2 (n=65 people).

Across the 4 sites, the aim is to collect information from 461 current MH workers. In addition, the study aims to collect feedback from 461 past MH workers and 461 non-MH workers. The overall projected sample size is 1383 participants.

### Study Procedures

#### Consent and Recruitment

Informed consent will be obtained from all participants prior to the completion of the survey or interview. Advertising of the project will be conducted with authorization by the principal investigator and Human Research Ethics Committee at each of the 4 sites for a period of 4 months. The study will be advertised through the distribution of emails to staff and posting information through staff intranet portals as well as by strategically placed physical posters. An email will be circulated via a broadcast that will include a link to the questionnaire, at which point they will be asked to provide informed consent to take part in the study. Current MH workers at each site will also be asked to forward the invitation to past MH colleagues who have left the public MH workforce. Participation in a qualitative interview will be offered through an indication of willingness to participate as indicated at the end of the web-based survey (see Multimedia Appendix 1) through expressions of interest in response to the flyers and through emails circulated via hospital broadcasts. There will be no costs associated with participating in this research project nor will participants be paid.

#### Survey Data Collection

The anonymous web-based survey was developed in conjunction with the steering committee and based on themes identified in the literature and preliminary interviews with senior hospital staff. In instances where there were no available validated measures, the research team created questionnaires for the purpose of this study based on the information available in the current literature and preliminary interviews. The survey will assess reasons for entering, staying in, or leaving public MH services, or reasons for having chosen not to work in the public MH sector. The survey will take approximately 20-25 minutes to complete. Participants will be asked to indicate their age, gender, ethnicity, highest level of education, current position, previous position (for past MH workers), discipline, hospital site, health service setting, current employment status, working hours preference, whether they are employed on a contract or an ongoing basis, duration in current position and duration working in MH (current and previous MH workers). The measures included in the web-based survey are detailed in Table 2. See Multimedia Appendix 2 for the full data collection instrument.

**Table 2:** Quantitative assessments in the survey.

Variable	Measure	Participants
Reasons for entering the public MH^a^ workforce	Eight key factors were identified in the literature plus an option to specify others. Participants will be asked to select all the responses that apply to their circumstances.	Current and previous MH workers
Attraction to the current organization	Sixteen key factors were identified in the literature plus an option to specify others. Participants will be asked to select all that apply to their circumstances.	All
Expectations and reality of working in public MH	The reality of working in public MH compared to expectations based on prior understanding of what a career in public MH looked like will be measured on a scale from 1 to 5, where a higher score is indicative of greater consistency between reality and expectations. This was developed for this study a review of the literature and themes identified in preliminary interviews with senior hospital staff. An additional open-ended question will assess reasons why expectations were or were not met.	Current and previous MH workers
Importance of MH care	Perceptions of the importance of MH care in comparison to other health care disciplines will be assessed by 2 statements that participants will be asked to respond to on a 5-point Likert scale (1=strongly disagree to 5=strongly agree). The statements were developed for this study based on a review of the literature and themes identified in preliminary interviews with senior hospital staff. These statements assess personal opinions and general health care worker beliefs.	All
Job readiness and career development	Job readiness (ie, sufficient training) and level of career development opportunities will be assessed by 6 statements that participants will be asked to respond to on a 5-point Likert scale (1=strongly disagree to 5=strongly agree). The statements were developed for this study based on a review of the literature and themes identified in preliminary interviews with senior hospital staff.	All
Workplace flexibility	Participants will be asked to rate the perceived level of their organization's workplace flexibility on a 10-point Likert scale, with 1 being not flexible at all and 10 being very flexible.	All
Resilience	The 9-item Employee Resilience Scale [16]. Responses will be measured on a 5-point Likert scale (1=strongly disagree to 5=strongly agree). Scores will be summed to produce a total score (range=9-45), whereby higher scores are indicative of greater levels of resilience in the workplace.	All
Burnout	Measured using the Oldenburg Burnout Inventory [17] which measures 2 dimensions of burnout: disengagement and exhaustion. Disengagement refers to distancing oneself from one’s work and having negative feelings towards one’s work or service recipients, while exhaustion refers to the depletion of physical and emotional energy.	All
Turnover intention	Measured by 3 statements: “I am actively looking for another job,” “As soon as I find another job, I will quit,” and “I often think about quitting my job” [18]. Each statement has three response options: (1) no, (2) unsure, and (3) yes. Overall turnover intention will be the average of the 3 responses, with higher scores indicating higher levels of turnover intention.	All
Current employment intention	Six statements developed through a review of the literature and anecdotal evidence will assess current employment intention. Participants will be asked to select all that apply to their circumstances.	All
Team leadership	Staff perceptions of team leadership and management qualities will be assessed by 5 statements measured on a 5-point Likert scale (1=strongly disagree to 5=strongly agree). These statements were developed for this study based on a review of the literature and themes identified in preliminary interviews with senior hospital staff. A total score was produced by summing all items (range=5-25), whereby higher scores were indicative of greater perceived leadership.	All that reported being in a nonleadership role
Understaffing	A 6-item measure of understaffing [19]. Participants are asked to respond to statements on a 5-point Likert scale (1=strongly disagree to 5=strongly agree). The measure provides subscale scores for manpower understaffing and expertise understaffing (range=3-15), whereby higher scores are indicative of greater understaffing. An additional question was developed for this study specifically related to staff shortages as a result of the COVID-19 pandemic, “COVID-19 has created additional staff shortages within our department/team.”	All
Job satisfaction	The 15-item Job Satisfaction Scale [20] with slight variations in wording to suit health care workers. Participants will be asked to respond on a 5-point Likert scale (1=very dissatisfied to 5=very satisfied). This measure provides 2 subscales of job satisfaction (extrinsic and intrinsic), as well as an overall score of job satisfaction (range=15-75), whereby higher scores are indicative of greater overall job satisfaction.	All
Workplace or occupational violence	A modified 9-item version of the World Health Organization Joint Program on Workplace Violence in the Health Sector questionnaire [21] will be used to assess workplace violence.	All
Moral injury	A modified 8-item version of the validated 10-item Moral Injury Symptom Scale-Health Professionals [22]. Each item is scored on a 5-point scale (1=strongly disagree to 5=strongly agree). Items are summed to create a total score (range=8-40), whereby higher scores are indicative of higher severity of moral injury. An additional item will also be used to assess the level of distress or impairment the moral injury has caused on their lives rated on a 5-point Likert scale (1=not at all to 5=extremely).	All
Reasons for leaving public MH workforce	Seven key factors were identified in the literature and preliminary interviews with senior hospital staff, plus an option to specify others. Participants will be asked to select all that apply to their circumstances.	Past MH workers
Reasons for not entering the MH workforce	Seven key factors were identified in the literature and preliminary interviews with senior hospital staff, plus an option to specify others. Participants will be asked to select all that apply to their circumstances.	Non-MH workers

^a^MH: mental health.

#### Qualitative Data Collection

A semistructured interview will be offered to those interested in providing more detailed information and recommendations for change within the field. This will enable a nuanced exploration of the most pertinent, empirically relevant themes associated with attraction, retention, and reasons for leaving or not entering the MH workforce. Interviews will be approximately 30-60 minutes in duration and conducted one-on-one with the research clinician either face-to-face or via a video call (ie, Teams, Microsoft Corp or Zoom, Zoom Technologies, Inc), depending on the participants’ preference. The qualitative component will cover the four areas: (1) participant demographics including age and gender; (2) questions regarding current employment information (eg, discipline and location, ie, rural or metro); (3) questions regarding the positive and negative aspects of working in the MH field, specifically related to attraction, recruitment, retention, or not entering the MH field (see Multimedia Appendix 2). Additionally, the themes identified in the literature (eg, burnout, understaffing, and moral injury) will be targeted through questioning to explore their relevance; and (4) any other factors perceived by the participant as relevant or important to attraction, recruitment, or retention.

#### Participant Withdrawal

Participants will be free to withdraw from the study without reason or explanation. Data collected will be retained unless otherwise specified by the participant. However, as the web-based survey is anonymous, it will not be possible for participants to withdraw their survey data.

### Statistical Analysis

#### Quantitative Data Analysis

Quantitative data analyses will be performed using SPSS (version 27; IBM Corp). Descriptive data will be presented using means, IQR, and percentages. Descriptive statistics that will be reported include discipline, employment status, hospital site location (ie, rural and metropolitan), health care setting (ie, inpatient and outpatient), duration of current position, and duration of working in health care. Groups will be categorized according to the variables listed as follows: (1) current MH worker, previous MH worker, and non-MH worker; (2) discipline (ie, nursing, medicine, allied health, and lived experience); (3) hospital site location (ie, regional or rural, metropolitan, using pooled rural and metropolitan data); and (4) health setting (ie, inpatient, community, and nonpatient facing).

Differences between groups on outcome measures will be compared using appropriate inferential tests. Regression analyses will also be conducted to determine predictors of retention (ie, job satisfaction) and turnover intention among the metropolitan workforce, and the regional or rural workforce.

#### Qualitative Data Analysis

Interviews will be recorded (with informed consent), transcribed verbatim, coded, and thematically analyzed to identify common emerging themes [23-25]. Transcripts will be continuously revisited, and the accuracy verified by listening to recordings and comparing them with the transcripts. Coding of data will be conducted using Excel (Microsoft Corp).

### Data Management

All electronic data will be stored securely via password protection within the primary hospital IT system in accordance with appropriate data management policies, with access available only to the research team. This includes electronic consent forms, interview transcripts, interview recordings, and survey data. All other investigators will be given access to the deidentified and pooled group data if required. Physical forms (ie, participant information and consent forms) collected during the interview or focus group process (if conducted face-to-face) will be stored in a secure, locked, cabinet at the primary hospital site and handling of the forms will be in accordance with hospital data management policies. Upon receiving informed consent to participate in the interview, the research clinician will assign participant IDs. All collected data will be stored in a deidentified manner using participant codes. The data will be reidentifiable using the code key, which will be stored separately from the data files. Only researchers employed on the project at the primary hospital site will have access to the code key. Information that is collected for this project will be stored for 7 years and then securely disposed of.

### Ethical Considerations

This study has been approved by the primary hospital Human Research Ethics Committee (HREC #008/22), and subsequently, governance approval was provided by the human research ethics committees at the other 3 sites. The findings from this project will be published and presented in a variety of forums, including peer-reviewed scientific journals and academic conferences. In all instances, information will be presented in a way that individuals and services cannot be identified achieved through presenting pooled group data only.

## Results

The project received funding in May 2021, and data collection commenced in July 2022. As of May 2023, we enrolled 539 participants in the web-based survey and 27 participants in the qualitative interview. Data analysis has commenced, with results expected to be published by December 2023.

## Discussion

### Summary

This project seeks to evaluate factors that influence attraction, recruitment, and retention within the public MH workforce in Victoria, Australia. The design of the project, based on previous research that illustrated both role and service/system-related difficulties, sought to build on current knowledge from within the Australian context and abroad. For instance, in its investigation of Victoria's Mental Health System, the Royal Commission discovered numerous obstacles that MH workers face, which may contribute to issues of retention. These included occupational burnout, violence and aggressive behavior from patients, frequent staff turnover, insufficient staffing levels, and, in some cases, a lack of management support [5]. Similarly, MH workers in Ghana cited job-related challenges such as lack of resources and workplace violence (eg, patient aggression and physical violence), as well as service- or system-based problems including rigid hierarchical organizational structure, limited opportunity for advancement, unclear pathways for progression, and lack of feedback or recognition regarding work performance, as significant issues related to retention [11]. These challenges and barriers in retention are potentially compounded by difficulties in attracting people to specific roles, and the MH sector more broadly. This may be reflected in the reported shortage of supply in appropriately qualified people applying for advertised positions and an inability to backfill roles where someone has been promoted [19,26].

Beyond aspects of the role that may contribute to problems with retention, previous studies have also demonstrated that due to the environment (ie, design not conducive to promoting safe practices for staff and patients and burdensome procedures), MH workers are at an increased risk of experiencing job-related stressors [27,28]. These stressors warrant further attention as they have been reported to contribute to decreased staff well-being and increased rates of attrition. For example, occupational burnout and workplace violence (both physical and psychological) have been reported among MH workers and are associated with reduced job satisfaction [27,28]. Job satisfaction has been reported to be lower in health care workers compared with workers in other professions [29]. Given the high-risk environment of MH, job satisfaction may be especially low in MH settings, although to date, this has not been investigated. Furthermore, job satisfaction has been linked to higher turnover intention (ie, the desire to leave one’s job) [27], which in turn may exacerbate staff shortages [29]. By exploring the relationships between these factors using both quantitative and qualitative methods, our study seeks to provide greater clarity relating to environmental aspects.

Although this study is somewhat limited by the participation of only a portion of the full workforce, one particular strength is the inclusion of both metropolitan and regional or rural services. This will allow investigation of similarities and differences and exploration of some of the unique challenges that may need to be considered in each setting. Furthermore, our study will build on others that have been conducted in regional and rural areas of Australia. Among these previous studies, factors specifically affecting attraction and retention have been explored in regional and rural areas of Queensland and New South Wales. Results indicate there are some unique differences when compared to metropolitan areas. For example, the distance and long travel times between towns that may not be adequately factored into workload expectations, limited transport options, reduced availability of services and amenities for workers and their families, less anonymity, perceptions of reduced opportunities for career advancement due to working in much smaller teams, and increased workload due to the expectation of having to educate other staff have all been reported as factors that influence staff turnover in regional and rural areas [30-32]. On the other hand, there may be particular strengths, and investigating the experiences of staff across both metropolitan and regional or rural settings will allow an examination of specific needs, and also how strengths may be leveraged.

To plan for the future and ensure that strategic initiatives are fit for purpose and aligned across employment settings, it is essential that a comprehensive evaluation of the life cycle of employment in MH settings be undertaken. Two factors should be considered. First, understanding what motivates people to work in public MH and second, how people subsequently perceive and experience their jobs, specifically what motivates them to stay or leave. Overall, this information will provide support for critical workforce planning to target the most pertinent factors identified by the current workforce, which will assist in ensuring the alignment of job supply with the demand required by the MH system, in order to create a sustainable MH workforce.

### Limitations

A limiting factor of this study is the potential for participant responses to inaccurately depict those of the population, a common issue within opt-in survey designs [33]. Moreover, the location may be considered a limitation given participants were only recruited from 4 health care services within Victoria, Australia. To address this, services were selected by the Victorian Department of Health, with the goal of capturing the most variety across the locations as possible. This study aimed to elucidate the challenges experienced by multidisciplinary teams specifically within Victorian public MH services. This means that generalizability beyond MH and Victoria may be hindered, and a potential future direction could be to replicate and extend this research more broadly for comparison and to increase the breadth of knowledge. The use of self-report measures is another limitation worth noting; and in an attempt to address this, this study selected to primarily use validated measures.

### Conclusions

With this study, we aim to broaden current knowledge regarding the recruitment and retention of multidisciplinary public MH care teams, specifically within Victoria, Australia. The findings of the study will be analyzed and used to inform necessary workforce planning, which supports the alignment of job supply with the demand required by the MH system into the future.

## Appendix

Multimedia Appendix 1 Interview expression of interest form.

Multimedia Appendix 2 Data collection instruments.

## Abbreviations

MH: mental health

## Data Availability

The data that support this study will be available upon reasonable request from the corresponding author.
