# Supplementary material for: Investigating Attraction and Retention of Staff Within Public Mental Health Services in Victoria, Australia: Protocol for a Mixed Methods Study
Source: JMIR Res Protoc. 2023 Oct 31;12:e48855. doi: 10.2196/48855 (PMC10646675; doi:10.2196/48855)
Supplement: Multimedia Appendix 1 [file resprot_v12i1e48855_app1.docx]

**Q1** Please provide your name and preferred contact details below, and a member of the research team will be in touch to organise a one-on-one interview to further explore the themes assessed in the survey related to attraction and retention of staff within the public mental health workforce.

Name __________________________

Email address _________________________

Phone number __________________________

**Q2** Please select the option that applies to you

- I am currently employed in the public mental health workforce
- I have previously been employed in the public mental health workforce
- I have never been employed in the public mental health workforce
