# Supplementary material for: Investigating Attraction and Retention of Staff Within Public Mental Health Services in Victoria, Australia: Protocol for a Mixed Methods Study
Source: JMIR Res Protoc. 2023 Oct 31;12:e48855. doi: 10.2196/48855 (PMC10646675; doi:10.2196/48855)
Supplement: Multimedia Appendix 2 [file resprot_v12i1e48855_app2.docx]

**Web-Based Survey**

**Q1** Please indicate which organisation you are currently/previously employed at.

**Q2** Now that you have read the Participant Information and Consent Form. Please indicate that you consent to participate by ticking the box below.

- I consent to participate

**Q3** Please select the option that applies to you

- I am currently employed in the public mental health workforce
- I have previously been employed in the public mental health workforce
- I have never been employed in the public mental health workforce

***Demographics***

**Q4** At which organisation were you most recently employed when you worked in mental health? *(past mental health workers)*

**Q5** Which mental health service do you currently work in? *(current mental health workers)*

**Q6** What department/health service did you work in as a mental health worker? *(past mental health workers)*

**Q7** What department/health service do you currently work in? *(past and non-mental health workers)*

**Q8** How old are you in years? _________________

- Prefer not to say

**Q9** Which gender do you identify with most?

- Man
- Woman
- Non-binary/gender diverse
- Prefer to self-describe (please specify) _________________
- Prefer not to say

**Q10** What ethnic group do you identify with?

- Aboriginal and/or Torres Strait Islander
- Anglo-Celtic (Caucasian)
- Central Asian (Sri-Lankan, Indian, Bangladeshi, Pakistan)
- East Asian (Chinese, Japanese, Korean, Mongolian, Taiwanese)
- Middle Eastern
- African
- Other European
- Other (please specify) __________________

**Q11** What is your highest level of completed education?

- Primary
- Secondary
- TAFE/diploma
- Undergraduate
- Masters/Doctorate/PhD
- Prefer to self-describe (please specify) __________________________

**Q12** What is/was your employment status?

- Full-time
- Part-time
- Casual
- Student

**Q13** Over the last 3-months, how many hours per week (on average) did you work? *(current or non-mental health worker and part-time or casual to Q12****)***

_______________________

**Q14** Over a period of 3-months, how many hours per week (on average) did you work while working in mental health? (*past mental health worker and part-time or casual to Q12*)

_______________________

**Q15** Are/were you on a fixed-term contract?

- Yes
- No

**If Yes to Q15, Q16** Would you (have) prefer(red) to be employed in a permanent position?

- Yes
- No

**If Yes to Q15, Q17** If you were offered a permanent position within your organisation, would you (have) accept(ed) it?

- Yes
- No

**Q18** In terms of your working hours, would you say:

- I would (have) prefer(red) to work more hours
- My working hours are/were about right
- I would (have) prefer(red) to work fewer hours

**Q19** If you answered that you would (have) prefer(red) to work more hours or fewer hours, please briefly explain the reasons below:

**Q20** On a scale of 1-10, how flexible is/was your workplace?

**Q21** What would a flexible role look like to you?

_________________________________________________

**Q22** What is/was your discipline?

- Nursing
- Medicine
- Psychiatry
- Psychology
- Occupational Therapy
- Social Work
- Lived Experience Work
- Counselling
- Administration
- Management
- Other (please specify) _____________________

**Q23** What is/was your job title? Please include your grade/level if applicable (e.g., Registered Nurse, Enrolled Nurse, Grade 1 Occupational Therapist, Clinical Psychologist, Admin Officer Grade 2, Service Manager Level 2)
*Reminder: this survey is anonymous, and data will only be reported as group pooled data*

_________________________________________________

- Prefer not to say

**Q24** What is the length of time (in years) you have spent in your current/previous role?

________________

**Q25** What is the length of time (in years) you have worked in public mental health? (*current and past mental health workers*)

________________

**Q26** Have you ever considered a career in mental health? (*non-mental health workers*)

- Yes
- No

***Attraction***

**Q27** What (previously) attracted you to working in the field of mental health? (Tick all that apply) *(current and past mental health worker)*

What would attract you to working in the field of mental health? (Tick all that apply) *(non-mental health worker)*

- Desire to help others
- Interest in mental health and human behaviour
- Desire to make a difference and do something worthwhile
- Ability to use skills and learned knowledge
- Motivated by lived experience (e.g., self/carer)
- Improve the mental health system
- Financial incentive
- Reduce stigma surrounding mental health
- Other (please specify) __________________________________________________

**Q28** How well does your experience of working in your current role meet the expectations you had of working in public mental health/the public sector prior to commencing your career? *(current and non-mental health worker)*

| Not at all |  | Somewhat |  | Very much |
| --- | --- | --- | --- | --- |
| 1 | 2 | 3 | 4 | 5 |
| ο | ο | ο | ο | ο |

**Q29** How well did your experience of working in your most recent mental health role meet the expectations you had of working in public mental health prior to commencing your mental health career? *(past mental health workers)*

| Not at all |  | Somewhat |  | Very much |
| --- | --- | --- | --- | --- |
| 1 | 2 | 3 | 4 | 5 |
| ο | ο | ο | ο | ο |

**Q30** If your experience has been/was different to what you expected, please briefly explain why this was the case.

________________________________________________________________

**Q31** What (previously) attracted you to working at your organisation? (Tick all that apply) (*current and past mental health workers*)

- I am a previous employee
- I completed a previous placement/pre-qualification experience within the organisation
- I have previously worked/collaborated with current employees within the organisation
- I or someone I know has previously received care within this organisation
- Recommended by someone I know
- It is a highly regarded organisation
- Career development opportunity
- Good promotion opportunities within the organisation
- Good culture and supportive work environment
- Innovative employer - novel practices/forward thinking
- The mission and values of the organisation aligned with my personal values
- Patient-oriented organisation
- Close to where I live
- Financial incentive
- Job security within the organisation
- Unable to find employment elsewhere
- Other (please specify) __________________________

**Q32** Why did you leave your role in public mental health? (Tick all that apply) *(past mental health workers)*

Please select from the list below contributions to your decision not to work in mental health (Tick all that apply) (*non-mental health worker*)

- Workplace violence
- Moral injury
- Occupational burnout
- Understaffing
- Lack of support from management/supervisors
- Lack of career progression opportunities
- Lack of training and development opportunities
- Other (please specify) ___________________________

**Q33** Please consider the following statements:

|  | Strongly disagree | Somewhat disagree | | | Neither agree nor disagree | | Somewhat agree | | Strongly agree | |
| --- | --- | --- | --- | --- | --- | --- | --- | --- | --- | --- |
|  | 1 | 2 | | | 3 | | 4 | | 5 | |
| 1. In my opinion, mental healthcare is at least equally as important as other healthcare disciplines | ο | | ο | ο | | ο | | ο | |  |
| 2. Healthcare workers in general believe that mental healthcare is at least equally as important as other healthcare disciplines | ο | | ο | ο | | ο | | ο | |  |

**Q34** Do you have any additional comments regarding the above statements?

________________________________________________________________

***Job Readiness and Career Development***

**Q35** Reflecting on your current role/most recent mental health role, please consider the following statements:

|  | Strongly disagree | Somewhat disagree | | | Neither agree nor disagree | | Somewhat agree | | Strongly agree | |
| --- | --- | --- | --- | --- | --- | --- | --- | --- | --- | --- |
|  | 1 | 2 | | | 3 | | 4 | | 5 | |
| 1. My tertiary training/education sufficiently prepared me to enter the mental health workforce | ο | | ο | ο | | ο | | ο | |  |
| 2. I have/had received the right level of ongoing training and opportunities to enhance my experience in order to undertake my role | ο | | ο | ο | | ο | | ο | |  |
| 3. I have/had mapped my career progression | ο | | ο | ο | | ο | | ο | |  |
| 4. I have/had access to further professional development | ο | | ο | ο | | ο | | ο | |  |
| 5. I have/had been given the opportunity to undertake leadership training | ο | | ο | ο | | ο | | ο | |  |
| 6. My qualifications allow(ed) me to work overseas in a similar role | ο | | ο | ο | | ο | | ο | |  |

**Q36** Do you have any additional comments or suggestions regarding job readiness or career development?

________________________________________________________________

***Employee Resilience Scale***

**Q37** Reflecting on your current role, please consider the following statements:

|  | Almost never | | |  | |  | |  | | Almost always | |
| --- | --- | --- | --- | --- | --- | --- | --- | --- | --- | --- | --- |
|  | 1 | | | 2 | | 3 | | 4 | | 5 | |
| 1. I effectively collaborate(d) with others to handle challenges at work | | ο | ο | | ο | | ο | | ο | |  |
| 2. I successfully manage(d) a high workload for long periods of time | | ο | ο | | ο | | ο | | ο | |  |
| 3. I resolve(d) crises competently at work | | ο | ο | | ο | | ο | | ο | |  |
| 4. I learn(t) from mistakes at work and improve(d) the way I do/did my job | | ο | ο | | ο | | ο | | ο | |  |
| 5. I re-evaluate(d) my performance and continually improve(d) the way I do/did my work | | ο | ο | | ο | | ο | | ο | |  |
| 6. I effectively respond(ed) to feedback at work, even criticism | | ο | ο | | ο | | ο | | ο | |  |
| 7. I seek/sought assistance at work when I need(ed) specific resources | | ο | ο | | ο | | ο | | ο | |  |
| 8. I approach(ed) managers when I need(ed) their support | | ο | ο | | ο | | ο | | ο | |  |
| 9. I use(d) change at work as an opportunity for growth | | ο | ο | | ο | | ο | | ο | |  |

***Oldenburg Burnout Inventory***

**Q38** Reflecting on your current role, please consider the following statements: *(current and non-mental health workers)*

|  | Strongly agree | Agree | Disagree | Strongly disagree |
| --- | --- | --- | --- | --- |
| 1. I always find new and interesting aspects in my work |  |  |  |  |
| 2. There are days when I feel tired before I arrive at work |  |  |  |  |
| 3. It happens more and more often that I talk about my work in a negative way |  |  |  |  |
| 4. After work, I tend to need more time than in the past in order to relax and feel better |  |  |  |  |
| 5. I can tolerate the pressure of my work very well |  |  |  |  |
| 6. Lately, I tend to think less at work and do my job almost mechanically |  |  |  |  |
| 7. I find my work to be a positive challenge |  |  |  |  |
| 8. During my work, I often feel emotionally drained |  |  |  |  |
| 9. Over time, one can become dis-connected from this type of work |  |  |  |  |
| 10. After working, I have enough energy for my leisure activities |  |  |  |  |
| 11. Sometimes I feel sickened by my work tasks |  |  |  |  |
| 12. After my work, I usually feel worn out and weary |  |  |  |  |
| 13. This is the only type of work that I can imagine myself doing |  |  |  |  |
| 14. Usually, I can manage the amount of my work well |  |  |  |  |
| 15. I feel more and more engaged in my work |  |  |  |  |
| 16. When I work, I usually feel energised |  |  |  |  |

**Q39** Reflecting on your most recent mental health role, please consider the following statements: *(past mental health workers)*

|  | Strongly agree | Agree | Disagree | Strongly disagree |
| --- | --- | --- | --- | --- |
| 1. I always found new and interesting aspects in my work |  |  |  |  |
| 2. There were days when I felt tired before I arrived at work |  |  |  |  |
| 3. It happened more and more often that I spoke about my work in a negative way |  |  |  |  |
| 4. After work, I tended to need more time than in the past in order to relax and feel better |  |  |  |  |
| 5. I could tolerate the pressure of my work very well |  |  |  |  |
| 6. I tended to think less at work and do my job almost mechanically |  |  |  |  |
| 7. I found my work to be a positive challenge |  |  |  |  |
| 8. During my work, I often felt emotionally drained |  |  |  |  |
| 9. Over time, one can become dis-connected from that type of work |  |  |  |  |
| 10. After work I had enough energy for my leisure activities |  |  |  |  |
| 11. Sometimes I felt sickened by my work tasks |  |  |  |  |
| 12. After my work, I usually felt worn out and weary |  |  |  |  |
| 13. This was the only type of work that I could imagine myself doing |  |  |  |  |
| 14. Usually, I could manage the amount of my work well |  |  |  |  |
| 15. I felt more and more engaged in my work |  |  |  |  |
| 16. When I worked, I usually felt energised |  |  |  |  |

***Turnover Intention***

**Q40** Reflecting on your current role, please consider the following statements: *(current and non-mental health workers)*

|  | No | Unsure | Yes | |  |
| --- | --- | --- | --- | --- | --- |
|  | 1 | 2 | 3 | |  |
| 1. I am actively looking for another job | ο | ο | | ο | |
| 2. As soon as I can find another job, I will leave | ο | ο | | ο | |
| 3. I often think about leaving my current role | ο | ο | | ο | |

**Q41** Reflecting on your current role, please consider the following statements: *(past mental health workers)*

|  | No | Unsure | Yes | |  |
| --- | --- | --- | --- | --- | --- |
|  | 1 | 2 | 3 | |  |
| 1. I was actively looking for another job | ο | ο | | ο | |
| 2. As soon as I can found another job, I left | ο | ο | | ο | |
| 3. I often thought about leaving my mental health role | ο | ο | | ο | |

**Q42** If you are considering leaving, what are the reasons for this? *(current and non-mental health workers)*

________________________________________________________________

**Q43** Is there anything that could be done that would change your mind about leaving your current role? *(current mental health workers)*

________________________________________________________________

**Q44** Is there anything that could have been done that would have changed your mind about leaving your mental health role? *(past mental health workers)*

________________________________________________________________

***Current Employment Intention***

**Q45** Reflecting on your current role, please consider the following statements (Tick all that apply) *(current and non-mental health workers)*

- I am in this role to gain experience
- I am in this role because I enjoy the work
- I plan to stay in this role for the remainder of my career
- I plan to stay in this role for at least 12 months
- I feel that I am overqualified for this role
- I feel that my expertise is utilised fully in this role
- None of the above

**Q46** Reflecting on your most recent mental health role, please consider the following statements (Tick all that apply) *(past mental health workers)*

- I was in the role to gain experience
- I was in the role because I enjoyed the work
- I initially planned to stay in the role for the remainder of my career
- I initially planned to stay in the role for at least 12 months
- I felt that I was overqualified for the role
- I felt that my expertise was utilised fully in the role
- None of the above

**Q47** Do you have any additional comments regarding your career intentions?

________________________________________________________________

***Team Leadership***

**Q48** Do/did you supervise a team?

- Yes
- No

**If No to Q48, Q49** Reflecting on your current role, please consider the following statements: *(current and non-mental health workers)*

|  | Never | | Sometimes | | About half the time | | | Most of the time | | Always | |
| --- | --- | --- | --- | --- | --- | --- | --- | --- | --- | --- | --- |
|  | 1 | | 2 | | 3 | | | 4 | | 5 | |
| 1. I feel supported by my manager/supervisor | | ο | | ο | | ο | ο | | ο | |  |
| 2. My manager/supervisor is a good role model | | ο | | ο | | ο | ο | | ο | |  |
| 3. I feel that I have a mentor who willingly shares their knowledge with me | | ο | | ο | | ο | ο | | ο | |  |
| 4. My manager/supervisor is available for me to ask for help or advice | | ο | | ο | | ο | ο | | ο | |  |
| 5. In my team, roles and responsibilities are clearly defined and fulfilled | | ο | | ο | | ο | ο | | ο | |  |

**If No to Q48, Q50** Reflecting on your most recent mental health role, please consider the following statements: *(past mental health workers)*

|  | Never | | Sometimes | | About half the time | | | Most of the time | | Always | |
| --- | --- | --- | --- | --- | --- | --- | --- | --- | --- | --- | --- |
|  | 1 | | 2 | | 3 | | | 4 | | 5 | |
| 1. I felt supported by my manager/supervisor | | ο | | ο | | ο | ο | | ο | |  |
| 2. My manager/supervisor was a good role model | | ο | | ο | | ο | ο | | ο | |  |
| 3. I felt that I had a mentor who willingly shared their knowledge with me | | ο | | ο | | ο | ο | | ο | |  |
| 4. My manager/supervisor was available for me to ask for help or advice | | ο | | ο | | ο | ο | | ο | |  |
| 5. In my team, roles and responsibilities were clearly defined and fulfilled | | ο | | ο | | ο | ο | | ο | |  |

**Q51** Do you have any additional comments to make regarding the above questions?

________________________________________________________________

***Understaffing***

**Q52** Reflecting on your current role, please consider the following statements: *(current and non-mental health workers)*

|  | Strongly disagree | Somewhat disagree | | | Neither agree nor disagree | Somewhat agree | | | Strongly agree | |
| --- | --- | --- | --- | --- | --- | --- | --- | --- | --- | --- |
|  | 1 | 2 | | | 3 | 4 | | | 5 | |
| 1. Our department/team needs more employees | ο | | ο | ο | | | ο | ο | |  |
| 2. There are not enough employees in our department/team to complete all required job tasks | ο | | ο | ο | | | ο | ο | |  |
| 3. If work goes undone in our department/team, it is primarily due to not having enough employees to do it | ο | | ο | ο | | | ο | ο | |  |
| 4. Our department/team is missing personnel with key knowledge and skills | ο | | ο | ο | | | ο | ο | |  |
| 5. Our department/team needs employees with different skills from those the group currently possesses | ο | | ο | ο | | | ο | ο | |  |
| 6. If work goes undone in our department/team, it is primarily due to not having someone who knows how to do it properly | ο | | ο | ο | | | ο | ο | |  |
| 7. COVID-19 has created additional staff shortages within our department/team. | ο | | ο | ο | | | ο | ο | |  |

**Q53** Reflecting on your most recent mental health role, please consider the following statements: *(past mental health workers)*

|  | Strongly disagree | Somewhat disagree | | | Neither agree nor disagree | Somewhat agree | | | Strongly agree | |
| --- | --- | --- | --- | --- | --- | --- | --- | --- | --- | --- |
|  | 1 | 2 | | | 3 | 4 | | | 5 | |
| 1. Our department/team needed more employees | ο | | ο | ο | | | ο | ο | |  |
| 2. There were not enough employees in our department/team to complete all required job tasks | ο | | ο | ο | | | ο | ο | |  |
| 3. If work went undone in our department/team, it was primarily due to not having enough employees to do it | ο | | ο | ο | | | ο | ο | |  |
| 4. Our department/team was missing personnel with key knowledge and skills | ο | | ο | ο | | | ο | ο | |  |
| 5. Our department/team needed employees with different skills from those the group currently possessed | ο | | ο | ο | | | ο | ο | |  |
| 6. If work went undone in our department/team, it was primarily due to not having someone who knew how to do it properly | ο | | ο | ο | | | ο | ο | |  |

**Q54** If you worked in mental health during the COVID-19 pandemic, please consider the following statement: *(past mental health workers)*

|  | Strongly disagree | Somewhat disagree | | | Neither agree nor disagree | Somewhat agree | | | Strongly agree | |
| --- | --- | --- | --- | --- | --- | --- | --- | --- | --- | --- |
|  | 1 | 2 | | | 3 | 4 | | | 5 | |
| COVID-19 created additional staff shortages within our department/team | ο | | ο | ο | | | ο | ο | |  |

**Q55** Do you have any additional comments regarding staffing levels?

________________________________________________________________

***Job Satisfaction Scale***

**Q56** Reflecting on your current role/most recent mental health role, please consider your level of dissatisfaction/satisfaction with the following statements:

|  | Extremely dissatisfied | | Somewhat dissatisfied | | Neither satisfied nor dissatisfied | | | Somewhat satisfied | | Extremely satisfied | |
| --- | --- | --- | --- | --- | --- | --- | --- | --- | --- | --- | --- |
|  | 1 | | 2 | | 3 | | | 4 | | 5 | |
| 1. The physical working conditions | | ο | | ο | | ο | ο | | ο | |  |
| 2. The freedom to choose your own method of working | | ο | | ο | | ο | ο | | ο | |  |
| 3. Your fellow workers and colleagues | | ο | | ο | | ο | ο | | ο | |  |
| 4. The recognition you get for good work | | ο | | ο | | ο | ο | | ο | |  |
| 5. Your immediate manager/supervisor | | ο | | ο | | ο | ο | | ο | |  |
| 6. The amount of responsibility you are given | | ο | | ο | | ο | ο | | ο | |  |
| 7. Your rate of pay | | ο | | ο | | ο | ο | | ο | |  |
| 8. Your opportunity to use your abilities | | ο | | ο | | ο | ο | | ο | |  |
| 9. Relationship between management and workers in your organisation | | ο | | ο | | ο | ο | | ο | |  |
| 10. Your chance for career progression | | ο | | ο | | ο | ο | | ο | |  |
| 11. The way your organisation is managed | | ο | | ο | | ο | ο | | ο | |  |
| 12. The attention paid to suggestions you make | | ο | | ο | | ο | ο | | ο | |  |
| 13. Your hours of work | | ο | | ο | | ο | ο | | ο | |  |
| 14. The amount of variety in your job | | ο | | ο | | ο | ο | | ο | |  |
| 15. Your job security | | ο | | ο | | ο | ο | | ο | |  |

***Workplace Violence***

**Q57** The following questions are potentially sensitive in nature and may be answered at your discretion. If you become upset or distressed as a result, please contact the research team and we will be able to arrange for counselling or other appropriate support.

Reflecting on your current role/most recent mental health role, please consider the following questions:

In the last 12 months, have you experienced workplace violence (i.e., physical violence, verbal abuse, bullying/mobbing, sexual harassment, and racial harassment)? *(current and non-mental health workers)*

- Yes
- No

**Q58** Did you experience workplace violence (i.e., physical violence, verbal abuse, bullying/mobbing, sexual harassment, and racial harassment)? *(past mental health workers)*

- Yes
- No

**If No to Q57/Q58, Q59** If you haven’t personally experienced workplace violence, have you witnessed incidents of workplace violence? *(current and non-mental health workers)*

- Yes
- No

**If No to Q57/Q58, Q60** If you haven’t personally experienced workplace violence, have you witnessed incidents of workplace violence? *(past mental health workers)*

- Yes
- No

*If Yes to Q57/58 or Q59/60, the following questions related to workplace violence were displayed to participants:*

**Q61** Which of the following types of workplace violence have/did you experience(d)? (Tick all that apply)

- Physical violence
- Verbal abuse
- Bullying/mobbing
- Sexual harassment
- Racial harassment

**Q62** Thinking of the worst incident of workplace violence that you have experienced, who perpetrated this violence?
*Reminder: this survey is anonymous, and data will only be reported as group pooled data*

- Patient
- Relatives of patient
- Staff member
- Management/supervisor
- External colleague/worker
- General public
- Other (please specify) __________________________________________________

**Q63** Do you consider your experiences of workplace violence to be typical of working in public mental health?

- Yes
- No
- Unsure

**Q64** What types of support did your employer or supervisor offer to provide you with following your experience of workplace violence? (Tick all that apply)

- Counselling
- Peer support program
- Opportunity to speak about/report it
- I didn't report it/tell anyone about it
- Other (please specify) __________________________________________________

**Q65** How satisfied were you with the way the incident of workplace violence was handled?

| Very dissatisfied | Somewhat dissatisfied | Neither satisfied nor dissatisfied | Somewhat satisfied | Very satisfied |
| --- | --- | --- | --- | --- |
| 1 | 2 | 3 | 4 | 5 |
| ο | ο | ο | ο | ο |

**Q66** Optional - Please explain why you were dissatisfied or satisfied with the way the incident of workplace violence was handled.

________________________________________________________________

**Q67** Do you have any suggestions in regards to how workplace violence and/or aggression is/was prevented and managed at your organisation?

________________________________________________________________

**Q68** Do you have any additional comments regarding workplace violence and/or aggression?

________________________________________________________________

**Q69** The following questions are potentially sensitive in nature and may be answered at your discretion. If you become upset or distressed as a result, please contact the research team and we will be able to arrange for counselling or other appropriate support.

Reflecting on your current role, please consider the following statements: *(current and non-mental health workers)*

|  | Strongly disagree | Somewhat disagree | Neither agree nor disagree | Somewhat agree | Strongly agree |
| --- | --- | --- | --- | --- | --- |
| 1. I feel betrayed by other health professionals whom I once trusted. |  |  |  |  |  |
| 2. I feel guilt over failing to save someone from being seriously injured or dying. |  |  |  |  |  |
| 3. I feel ashamed about what I’ve done or not done when providing care to my patients. |  |  |  |  |  |
| 4. I am troubled by having acted in ways that violated my own morals or values. |  |  |  |  |  |
| 5. Most people with whom I work as a health professional are trustworthy. |  |  |  |  |  |
| 6. I have a good sense of what makes my life meaningful as a health professional. |  |  |  |  |  |
| 7. I have forgiven myself for what has happened to me or others whom I have cared for. |  |  |  |  |  |
| 8. All in all, I am inclined to feel that I’m a failure in my work as a health professional. |  |  |  |  |  |

**Q70** Reflecting on your most recent mental health role, please consider the following statements: *(past mental health workers)*

|  | Strongly disagree | Somewhat disagree | Neither agree nor disagree | Somewhat agree | Strongly agree |
| --- | --- | --- | --- | --- | --- |
| 1. I felt betrayed by other health professionals whom I once trusted. |  |  |  |  |  |
| 2. I felt guilt over failing to save someone from being seriously injured or dying. |  |  |  |  |  |
| 3. I felt ashamed about what I’d done or not done when providing care to my patients. |  |  |  |  |  |
| 4. I was troubled by having acted in ways that violated my own morals or values. |  |  |  |  |  |
| 5. Most people with whom I worked as a health professional are trustworthy. |  |  |  |  |  |
| 6. I had a good sense of what makes my life meaningful as a health professional. |  |  |  |  |  |
| 7. I had forgiven myself for what had happened to me or others whom I have cared for. |  |  |  |  |  |
| 8. All in all, I was inclined to feel that I was a failure in my work as a health professional. |  |  |  |  |  |

**Q71** Do/did the feelings you indicated above cause you significant distress or impair your ability to function in relationships, at work, or other areas of life important to you? In other words, if you indicated any problems above, how difficult have/did these problems made/make it for you to do your work, take care of things at home, or get along with other people?

| Not at all | Mild | Moderate | Very much | Extremely |
| --- | --- | --- | --- | --- |
| 1 | 2 | 3 | 4 | 5 |
| ο | ο | ο | ο | ο |

**Q72** Do you have any additional comments regarding the above statements?

________________________________________________________________

**Qualitative Interview**

***Current Mental Health Worker***

1. Following the completion of your training, how well equipped did you feel to enter a role within public mental health? *(Prompts used for specific details and examples).*
2. Based on your experiences, what are some of the best things about working in public mental health? *(Prompts if required: e.g., the team, environment, satisfaction of helping, development, remuneration).*
3. What are some of the most challenging aspects of your role and what is their impact? *(Prompts if required: e.g., burnout, poor leadership, understaffing, feeling unsafe, unsupported, bullying, low autonomy, moral injury).*
4. Why do you choose to stay? *(Prompts if required: e.g., intrinsic/extrinsic motivators – could be connected to Q4).*
5. How do you handle the demands of working in mental health? (*Prompts if required: e.g., other team member’s/manager’s support, sense of making a difference, good coping strategies*).
6. How would you describe a good leader in public mental health?
7. If you could change 2-3 things to improve your experience in your role, what would they be? *(Prompts if required: e.g., people, environment, profession, the work itself)*.
8. If you think about planning for the future, what do you think that the public mental health system needs the most? *(Is this something that you believe is being worked towards?)*.
9. Have you noticed any changes since the COVID-19 pandemic?

***Past Mental Health Worker***

1. Based on your experiences, what are some of the best things about working in public mental health? *(Prompts if required: e.g., the team, environment, satisfaction of helping, development, renumeration).*
2. What were some of the most challenging aspects? *(Prompts if required: e.g., burnout, poor leadership, under staffing, feeling unsafe, unsupported, bullying, low autonomy).*
3. Why did you choose to leave Public MH? *(Prompts if required: e.g., people, environment, profession, the work itself).*
4. How would you describe a good leader/good leadership in public mental health?
5. If you think about planning for the future, what do you think that the public mental health system needs the most? *(Is this something that you believe is being worked towards?).*

***Non-Mental Health Worker***

1. Why did you select the discipline/area that you are currently working in? (as opposed to MH)
2. Based on what you have heard from others/what you know, what are some of the best things about working in public mental health?
3. What are some of the most challenging aspects? *(Prompts if required: e.g., burnout, poor leadership, under staffing, feeling unsafe, unsupported, bullying, low autonomy).*
4. Have you ever considered a role in public mental health? *(Prompts to elaborate on answer where appropriate.)*
5. If you think about planning for the future, what do you think that the public mental health system needs the most? *(Is this something that you believe is being worked towards?)*
